# Primary Angiosarcoma of the Spleen: Rare Diagnosis with Atypical Clinical Course

**DOI:** 10.1155/2016/4905726

**Published:** 2016-10-27

**Authors:** Filip Kohutek, Ladislav Badik, Branislav Bystricky

**Affiliations:** ^1^Department of Oncology, Faculty Hospital Trencin, Trencin, Slovakia; ^2^Department of Radiology, Faculty Hospital Trencin, Trencin, Slovakia

## Abstract

Primary angiosarcoma of the spleen is a rare diagnosis with poor prognosis. Morphologically, it demonstrates conventional blood vessel differentiation. We present a case of 65-year-old female who underwent radical splenectomy for primary angiosarcoma of the spleen. After three-year disease-free interval, she was diagnosed with bone-only metastatic disease. Palliative radiotherapy and bisphosphonates kept her disease reasonably stable for another four years. After development of lung metastases, six cycles of single agent doxorubicin kept her progression-free for six years. Upon further progression in lungs, thirteen years after original diagnosis, lung biopsy confirmed metastatic splenic angiosarcoma in the lungs. She started weekly paclitaxel chemotherapy. Although splenic angiosarcoma generally carries grave prognosis, some patients may enjoy prolonged periods of disease stabilization. Durable benefit can be achieved in some patients with multimodality management. We review the literature focusing on systemic treatment for this rare tumor.

## 1. Introduction

Soft tissue sarcomas (STS) represent heterogeneous group of malignant tumors originating in mesenchymal tissue. These malignancies are rare and their treatment is usually centralized into large treatment centers. In the United States, estimated number of new cases of STS for 2016 is 12,310 and estimated number of deaths is 4,900 [1]. In Europe, average incidence of this group of malignancies is 4-5 patients/100,000 person-years [2]. According to WHO, STS can be classified into 40 morphological entities. There is a clear need for grouping of those numerous entities into larger groups in clinical practice as was done in non-Hodgkin's lymphomas.

Angiosarcomas contribute by less than 1% to all cases of STS. Malignant cells of angiosarcoma are derived from endothelial cells. Skin and visceral organs are mostly affected. On the contrary, only few cases of heart or large vessels involvement have been described. According to study of 98 cases by Shon et al. [3], there was no difference in prognosis between skin and visceral angiosarcoma.

However, the results of retrospective study of Fury et al. [4] showed that primary site of angiosarcoma actually had impact on its prognosis; better median survival (43.1 m versus 27.6 m) was observed in superficial (skin) angiosarcomas.

Herein we present a case of female patient with primary splenic angiosarcoma. Despite development of stage IV disease, long-time survival and good quality of life could be observed.

## 2. Case Report

**Figure 1:**
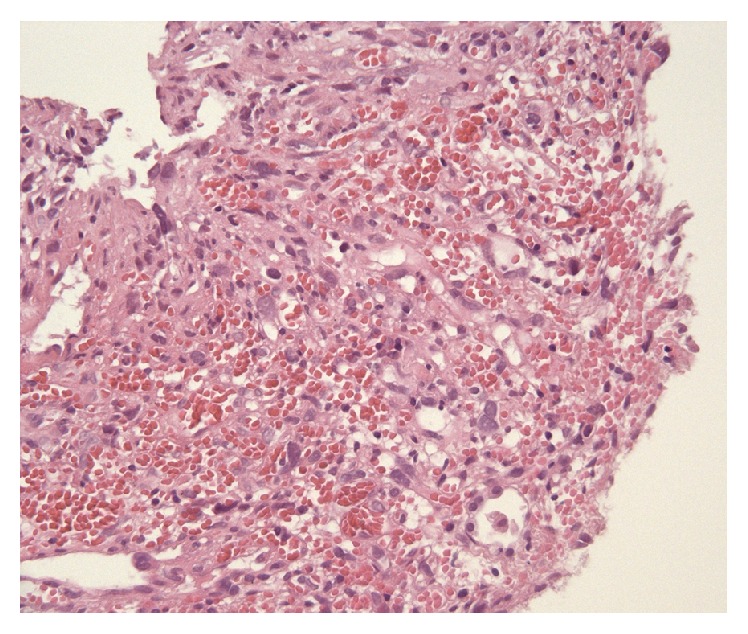
Primary splenic angiosarcoma. Staining with hematoxylin and eosin. 20-fold magnification.

**Figure 2:**
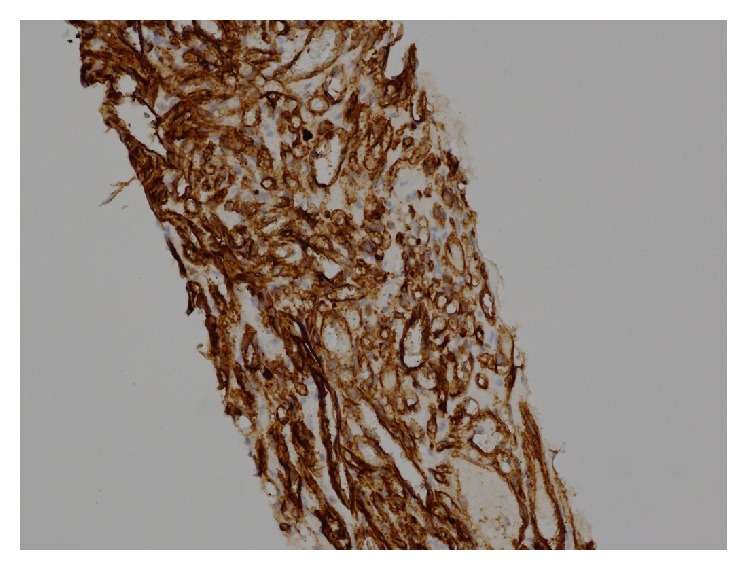
Primary splenic angiosarcoma. Immunohistochemical CD34 staining. 20-fold magnification.

**Figure 3:**
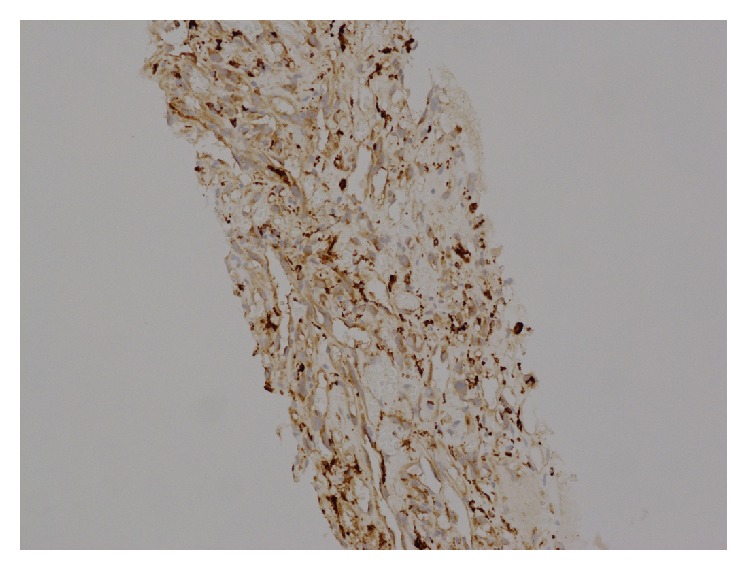
Primary splenic angiosarcoma. Immunohistochemical CD68 staining. 20-fold magnification.

**Figure 4:**
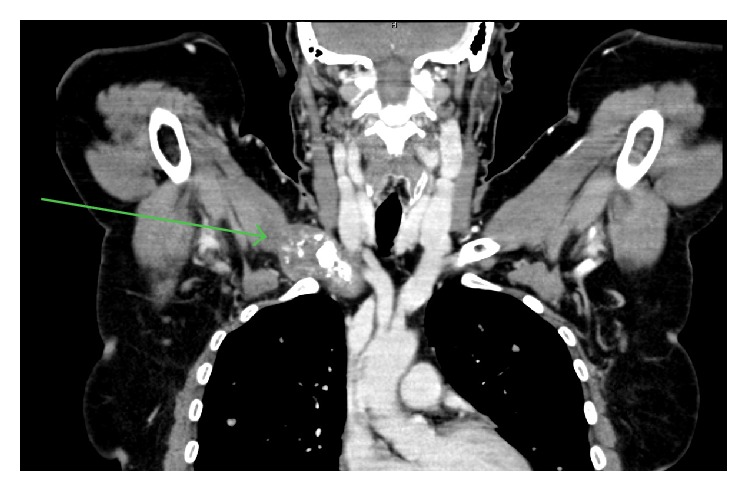
CT scan of thorax (multiplanar reconstruction). The green arrow points to osteolytic metastasis of right clavicle.

65-year-old female patient was admitted to Faculty Hospital Trencin, Department of Surgery, in January 2003 with palpable abdominal mass in left hypochondrium. The patient was only complaining about mild abdominal pain. Her past medical history was unremarkable, and she did not have any known risk factors for angiosarcoma. Abdominal mass was detected by patient's general practitioner during regular health exam. CT was performed and large hypervascularised splenic tumor at size of 5 × 4 cm was detected. No metastases were present at the CT scan. Mild anaemia (haemoglobin 11 g/dL), leucocytosis (13.10 × 10^9^/L), and thrombocytosis (507 × 10^9^/L) could be observed in complete blood count. Biochemistry showed no abnormalities. Due to increased vascularity of tumor and possibility of seeding in localized malignancy, no CT-guided biopsy was performed. Subsequently, radical splenectomy was performed and definitive diagnosis of low-grade primary splenic angiosarcoma was made (Figures 1, 2, and 3). No adjuvant treatment was indicated due to low-grade disease and complete resection; routine follow-up was initiated. Three years later, the patient was complaining about 1-month lasting pain localized to upper thoracic spine and irradiating to neck and head. CT and bone scan was performed and multiple bone metastases (sacrum, left 11th rib, right scapula, right humerus, and right clavicle) were detected (Figure 4). Laboratory results showed no significant abnormalities in hematological or biochemical parameters. Palliative radiotherapy at dose of 20 Gy to right arm and clavicle was administered due to painful nature of these metastatic sites. Bisphosphonates were introduced into treatment as well. Palliative chemotherapy was proposed, but it was refused by patient. After 12 months, new metastatic deposit appeared at right sternoclavicular joint. Another course of palliative radiotherapy (20 Gy) was administered to affected site. Patient's attitude towards palliative chemotherapy did not change. However, during subsequent three years, no progression was detected on imaging.

**Figure 5:**
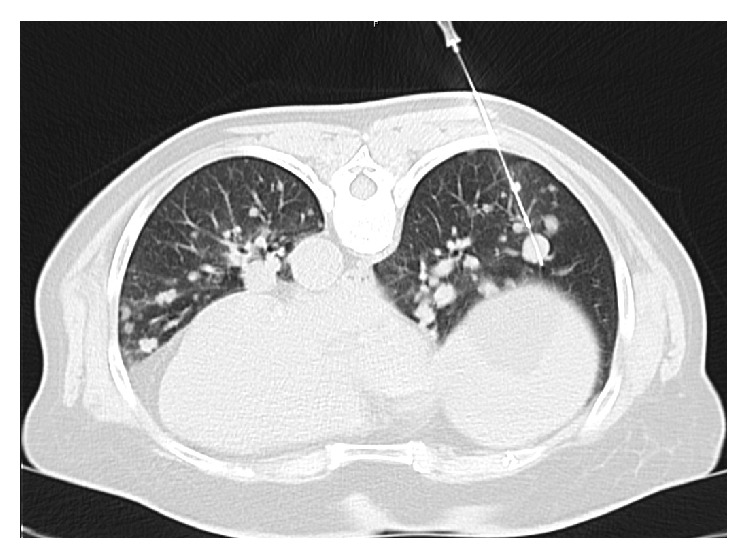
CT-guided needle biopsy of pulmonary metastasis of splenic angiosarcoma (20 G Trucut needle).

**Figure 6:**
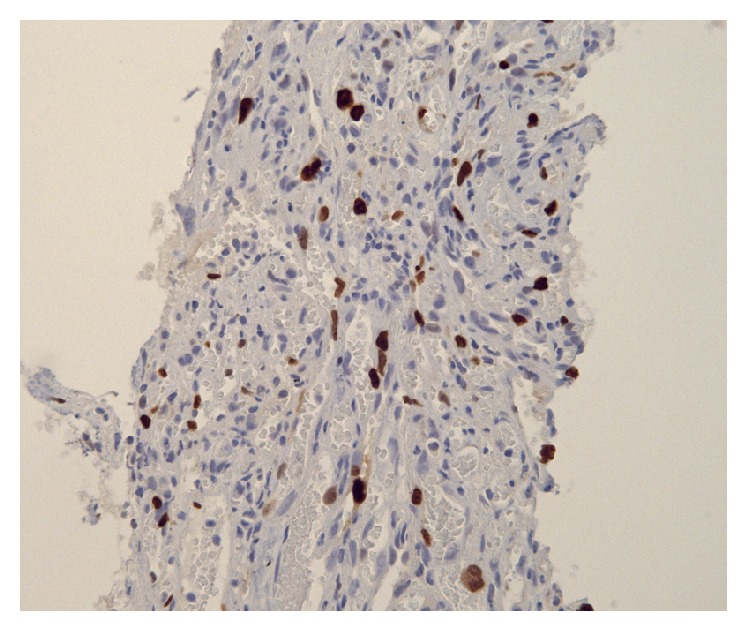
Pulmonary metastasis of splenic angiosarcoma. Immunohistochemical MIB1 staining. 20-fold magnification.

Unfortunately, in 2010 new metastases in liver and axial skeleton were detected. With patient's approval, 6 cycles of palliative chemotherapy (doxorubicin 60 mg/m^2^ triweekly) were given. During subsequent follow-up, no progression of disease was detected until 2016, when progression of metastatic lung lesions was noted. CT directed lung biopsy was performed (Figure 5). The result confirmed angiosarcoma metastasis. Vascular spaces were lined by atypical, highly pleomorphic cells with high nuclear to cytoplasm ratio. Intraluminally, occasional erythrocytes were seen. These cells were immunohistochemically positive for CD31, confirming vascular tumor arising from endothelial cells. MIB1 proliferation was 15% (Figure 6). At present, second-line chemotherapy (paclitaxel weekly) is ongoing.

## 3. Discussion 

Exposure to radiation and lymphedema [5] and exposure to chemicals such as thorium, arsenic, and vinyl chloride are mostly recognized risk factors of angiosarcoma [6]. Retrospective study of Fayette et al. [5] showed that breast angiosarcoma contributes by 35% to all angiosarcomas. Whereas postoperative radiation following quadrantectomy or lumpectomy increases relative risk (RR) of angiosarcoma development more than 1,000 times, it can be concluded that breast angiosarcoma development is late adverse effect of adjuvant radiotherapy.

Splenic vascular tumors are currently classified into three broad categories: tumors arising from endothelial lining of blood vessels, lymphatic endothelium, and specialized endothelium (littoral/sinusoidal cells) with morphologic overlap between them [7].

It is presumed that splenic angiosarcoma originates from vascular endothelium of splenic sinuses [8]. It was described for the first time in 1879 by Langhans. Until now, only 300 cases of splenic angiosarcoma have been reported. Still, it is the most frequent nonlymphoid primary cancer of the spleen. Worldwide incidence of this disease reaches 0.14–0.25/million persons.

Natural course of this disease is usually characterized by rapid progression and unfavorable outcome for the patient. The most common symptom is abdominal pain and fullness with splenomegaly, anemia, and thrombocytopenia. Fatigue, weight loss, and fever are common systemic complaints of patients. Life threatening rupture of spleen is the first manifestation of this malignancy in numerous cases. Early appearance of metastases is typical; liver, lung, lymph nodes, and gastrointestinal system are preferred sites of metastases [9]. Median survival is only 5 months [10].

Considering rarity of this disease, no international guidelines are available for diagnosis and treatment of splenic angiosarcoma. If neoadjuvant treatment of intraabdominal sarcoma is intended, biopsy is indicated [11]. However, biopsy of highly vascularized spleen is considerably risk-bearing and is not usually performed. On CT scan, splenic angiosarcoma can present as heterogeneous mass with central hypoattenuation, except in acute bleeding, where high attenuation is observed. MRI shows hypointensive masses on T1 and high signal on T2 if necrosis or bleeding is present. Both imaging modalities can reveal splenomegaly and occasional splenic rupture with hemoperitoneum.

Radical surgical treatment (splenectomy) remains the only curative modality in the treatment of splenic angiosarcoma. Patients should undergo vaccination prior to or immediately after splenectomy in order to prevent severe bacterial infections. Splenectomy also provides tissue for histological examination. Although postoperative radiotherapy is associated with decreased local recurrence rate, it has not improved overall survival in intraabdominal sarcomas [12].

In unresectable stages, downstaging preceding surgery by neoadjuvant chemotherapy, radiotherapy, or chemoradiation can be attempted. If neoadjuvant therapy does not achieve sufficient down staging, treatment is palliative. Taxanes with or without gemcitabine are regarded as most effective in treatment of angiosarcoma.

In general, taxanes are considered to be effective in angiosarcoma but not in other soft tissue sarcomas. However, only limited data are available to support this statement.

Efficacy of weekly paclitaxel in advanced angiosarcoma was studied in phase II trial of French Sarcoma Group [13]. 30 patients with metastatic or locally advanced unresectable angiosarcoma were included into prospective study. Paclitaxel was administered at dose of 80 mg/m^2^ on days 1, 8, and 15 of 4-week-long cycle. Taxane-based therapy achieved impressing results. Two-month-PFS was 74% and 4-month-PFS was 45%. Median time to progression (TTP) was 4 months and median overall survival (OS) was 8 months. Survival rates did not differ significantly in pretreated and chemotherapy-naive patients. Seven patients suffered from grade 3 or 4 toxicity and 1 patient died due to severe thrombocytopenia. Authors stated that paclitaxel-based chemotherapy of advanced angiosarcoma brought significant clinical benefit with acceptable toxicity.

Hirata et al. [14] published retrospective study of 41 patients with metastatic angiosarcoma. Patients were divided into 3 groups according to given treatment: taxane, nontaxane, and best-supportive care. Taxane-based chemotherapy achieved significant prolongation of PFS and OS compared to non-taxane-based chemotherapy and best-supportive care. Authors suggest that taxane-based chemotherapy may be more effective than nontaxane therapy in this setting.

The search for predictive factors of taxane chemotherapy brought first results. Expression of transducin-like enhancer of split 3 (TLE3) has been associated with better outcomes of taxane therapy [3].

As mentioned above, angiosarcoma is a malignancy originating from vascular endothelium and proangiogenic factors, such as vascular endothelial growth factor (VEGF), playing an important role in its pathogenesis. Sunitinib has been approved for imatinib-refractory GIST and in advanced renal-cell carcinoma utilizing its antiangiogenic effect. So far, no randomized controlled trials are available with sunitinib in treatment of advanced angiosarcoma. Several case reports have been published. Yoo et al. [15] published a case report of older woman with chemotherapy-refractory advanced angiosarcoma of retroperitoneum. Whereas patient's worsening performance status did not allow the use of another chemotherapy regimen, sunitinib was used as a last resort. Treatment with sunitinib was associated with impressing tumor control. Volume doubling time (VDT) of tumor based on serial CT scans was calculated. Compared to previous chemotherapy, VDT value on sunitinib was significantly longer (145 days on sunitinib versus 16 days on paclitaxel and 66 days on doxorubicin).

Sorafenib is a tyrosine-kinase inhibitor targeting several tyrosine-kinases such as BRAF, vascular endothelial growth factor receptor (VEGFR), and others [16]. Efficacy of sorafenib in angiosarcoma was studied in phase II trial [17] of French Sarcoma Group. In this trial, patients were divided into two groups: those with superficial angiosarcoma and those with visceral angiosarcoma. Sorafenib showed insufficient antitumor activity in both groups. Nine-month PFS was 3.8% in patients with superficial angiosarcoma and 0% in patients with visceral sarcomas. The trial was halted prematurely due to unsatisfying results. Another multicenter phase II trial of Maki et al. [18] studied activity of sorafenib against various histological subtypes of sarcomas. In this trial, sorafenib was administered to patients with advanced sarcoma at the dose of 400 mg twice a day. 14% of patients treated with sorafenib had a partial response (response rate 14%). Median PFS was 3.2 months and median OS was 14.3 months. However, in the angiosarcoma subgroup, PFS was 3.8 months and OS was 14.9 months. Contrary to previous French phase II trial, sorafenib proved to be efficient in treatment of angiosarcoma in this US trial. However, sorafenib showed no antitumor activity in other histological subtypes of sarcoma although it had minor activity against leiomyosarcoma.

Several case reports suggest that pazopanib may be effective in angiosarcoma treatment [19–21]. Phase III trial PALETTE by van der Graaf et al. [22] confirmed efficacy of pazopanib in advanced STS treatment in pretreated patients. Patients were divided into 2 arms: pazopanib monotherapy arm and placebo arm. Pazopanib treatment brought significant improvement of survival rates (PFS benefit of 3 months and OS benefit of 1.8 months). However, no relevant subanalysis for angiosarcoma is available in this trial.

Addition of bevacizumab to taxane-based chemotherapy was studied in randomized phase II trial of Ray-Coquard et al. [23]. Patients were treated with paclitaxel weekly (90 mg/m^2^) or combination of paclitaxel (90 mg/m^2^) weekly and bevacizumab (10 mg/kg biweekly). In the combination arm, six courses of bevacizumab were given and followed by maintenance therapy with bevacizumab (15 mg/kg triweekly) until progression. Primary endpoint was 6-month PFS. Six-month PFS rate was 54% in paclitaxel only arm and 57% in paclitaxel and bevacizumab arm. Median overall survival reached 19.5 months in paclitaxel only arm and 15.9 months in the combination arm. Authors concluded that addition of bevacizumab to paclitaxel failed to improve survival rates and recommended no further clinical investigation on this combination in advanced angiosarcoma treatment.

According to ESMO clinical practice guidelines, gemcitabine and docetaxel are an alternative to taxane-only chemotherapy of angiosarcoma. Efficacy of combination therapy consisting of gemcitabine, docetaxel, and bevacizumab (GDB) in treatment of soft tissue sarcomas was studied in phase II trial of Dickson et al. [24]. In the angiosarcoma subgroup, treatment with GDB combination resulted in at least partial responses in 60% of patients. Authors concluded that GDB combination represents a viable option in treatment of angiosarcomas.

Thalidomide is a drug well known for decades. It is presumed that thalidomide may play role in inhibition of angiogenesis, tumor necrosis factor inhibition, and immune system stimulation. However, exact effect of thalidomide on particular malignancies remains unclear [25]. Unfortunately, no RCTs but only few case reports are available on efficacy of thalidomide in advanced angiosarcoma treatment. Raina et al. [26] reported complete response in patient with secondary angiosarcoma of breast treated with thalidomide. In this case, thalidomide was chosen due to patient's advanced age (75 years) and questionable efficacy of chemotherapy. Complete response was achieved at the dose of 200 mg daily within 4 weeks. Adverse effects of thalidomide led to dose reduction (50 mg/daily) which resulted in disease recurrence after 8 weeks of dose reduction. Reestablishment of initial dose of 200 mg/daily led to disease stabilization.

Alvarado-Miranda et al. [27] published a case of 41-year-old woman with no history of malignancy with primary locally advanced angiosarcoma of left breast with axillary lymph node involvement. After neoadjuvant chemotherapy (four cycles of doxorubicin 50 mg/m^2^/cisplatin 75 mg/m^2^ in combination with thalidomide at dose of 200 mg daily followed by combined chemotherapy of paclitaxel 80 mg/m^2^/cisplatin 30 mg/m^2^ with thalidomide at the dose of 200 mg/daily), complete pathological response was observed. Subsequently, mastectomy and axillary lymph node dissection were performed and 6-month-long disease-free survival was observed.

These case reports suggest efficacy of thalidomide in angiosarcoma treatment. However, further investigation, preferably RCTs, is required to confirm these results.

In 2015, Banavali et al. [28] reported a case of 69-year-old woman with relapsing metastatic skin angiosarcoma treated with a combination of metronomic chemotherapy and nonselective beta-blocker propranolol. The patient was treated with low doses of cyclophosphamide and etoposide along with nonsteroid anti-inflammatory drug (NSAID) celecoxib and propranolol. The treatment was delivered according to particular schedule. Propranolol at the dose of 20 mg twice a day was delivered since the malignancy was positive for beta-adrenergic receptor. The treatment was associated with complete response after 2 cycles of treatment. Progression occurred 20 months after initiation of metronomic treatment. Patient died 7 months later. Authors underlined the fact that metronomic treatment proved to be innovative yet inexpensive therapy, the outcomes of which can be compared to more conventional regimens and modern and expensive targeted therapies.

Although angiosarcoma is generally considered to be rapidly progressive, our patient is surviving 13 years after being diagnosed with this malignancy. To our knowledge, this is one of the longest survivals of adult splenic angiosarcoma reported in literature, in addition to one patient reported by Hsu et al. [29]. Notably, metastatic spread occurred only 3 years (2006) after initial diagnosis (2003). It is unusual that systemic treatment had not been administered until 4 years (2010) later. No progression was detected during this 4-year-long interval in metastatic disease. Finally, first-line chemotherapy resulted in 6-year-long progression-free survival.

Several cases of long-term survival of patients with metastatic angiosarcoma have been reported. In 1988, Rosner [30] published case report of two patients with angiosarcoma of breast surviving more than 10 years after mastectomy and vinblastine-based adjuvant chemotherapy.

Hara et al. [31] presented a case of 48-year-old woman with localized splenic angiosarcoma. Splenectomy was performed and 3 courses of adjuvant chemotherapy were administered. CHOP regimen (cyclophosphamide, doxorubicin, vincristine, and prednisolone) was chosen in this setting. Moreover, due to poor prognosis, high-dose chemotherapy and autologous peripheral blood stem-cell transplantation (auto-PBSCT) were performed. This radical treatment brought 6-year-long complete remission. Afterwards, rapid course of recurring disease with massive metastatic spread and worsening of performance status led to patient's death within several months.

Finally, retrospective study of Fayette et al. [5] showed that survival rates in angiosarcoma are influenced by primary site of angiosarcoma. If angiosarcoma originates in visceral organs such as liver, spleen, bone, or heart, worse survival rates can be observed. On the other side, authors reported that significant proportion of patients with initially metastatic disease survived beyond 8 years after being diagnosed with angiosarcoma.

It is obvious that significant proportion of patients with advanced angiosarcoma may experience long-term survivorship. This long-term survivorship might be determined by unrecognized prognostic factors. However, it is unclear whether selection of systemic treatment contributes to this long-term survivorship. Without identifying these prognostic factors, this question remains unanswered.

## 4. Conclusion

Optimal treatment for primary splenic angiosarcoma remains unknown. ESMO and NCCN provide guidelines based on limited data and treatment outcomes for primary splenic angiosarcoma are poor in most patients. However, as suggested above, strict selection of patients based on prognostic and predictive factors and subsequent tailored therapy might bring impressive results. The research should focus on identifying those prognostic and predictive factors. However due to rarity of this disease, this task may be a question of long-term research.

## References

[1] American Cancer Society (2016). *Cancer Facts and Figures 2016*.

[2] The ESMO/European Sarcoma Network Working Group (2014). Soft tissue and visceral sarcomas: ESMO Clinical Practice Guidelines for diagnosis, treatment and follow-up. *Annals of Oncology*.

[3] Shon W., Jenkins S. M., Ross D. T. (2011). Angiosarcoma: a study of 98 cases with immunohistochemical evaluation of TLE3, a recently described marker of potential taxane responsiveness. *Journal of Cutaneous Pathology*.

[4] Fury M. G., Antonescu C. R., Van Zee K. J., Brennan M. F., Maki R. G. (2005). A 14-year retrospective review of angiosarcoma: clinical characteristics, prognostic factors, and treatment outcomes with surgery and chemotherapy. *Cancer Journal*.

[5] Fayette J., Martin E., Piperno-Neumann S. (2007). Angiosarcomas, a heterogeneous group of sarcomas with specific behavior depending on primary site: a retrospective study of 161 cases. *Annals of Oncology*.

[6] Gupta A., Patnaik M. M., Naina H. V. (2014). Angiosarcoma of the prostate gland following brachytherapy for prostatic adenocarcinoma. *Current Urology*.

[7] Kutok J. L., Fletcher C. D. M. (2003). Splenic vascular tumors. *Seminars in Diagnostic Pathology*.

[8] Despoina M., Dionysios D., Georgios A., Konstantinos S., Efstratios K., Adamantia Z.-S. (2014). Primary angiosarcoma of the spleen: an oncological enigma. *Case Reports in Oncological Medicine*.

[9] Xu B., Xie X., Zhou X., Zhai M., Yang W. (2015). Spontaneous rupture of primary splenic angiosarcoma: a case report. *Oncology Letters*.

[10] Duan Y.-F., Jiang Y., Wu C.-X., Zhu F. (2013). Spontaneous rupture of primary splenic angiosarcoma: a case report and literature review. *World Journal of Surgical Oncology*.

[11] National Comprehensive Cancer Network https://www.nccn.org/professionals/physician_gls/pdf/sarcoma.pdf.

[12] Jones J. J., Catton C. N., O'Sullivan B. (2002). Initial results of a trial of preoperative external-beam radiation therapy and postoperative brachytherapy for retroperitoneal sarcoma. *Annals of Surgical Oncology*.

[13] Penel N., Bui B. N., Bay J.-O. (2008). Phase II trial of weekly paclitaxel for unresectable angiosarcoma: the ANGIOTAX Study. *Journal of Clinical Oncology*.

[14] Hirata T., Yonemori K., Ando M. (2011). Efficacy of taxane regimens in patients with metastatic angiosarcoma. *European Journal of Dermatology*.

[15] Yoo C., Kim J.-E., Yoon S.-K. (2009). Angiosarcoma of the retroperitoneum: report on a patient treated with sunitinib. *Sarcoma*.

[16] Sleijfer S. (2012). Phase II studies in soft tissue sarcoma: time for reappraisal. *The Oncologist*.

[17] Ray-Coquard I., Italiano A., Bompas E. (2012). Sorafenib for patients with advanced angiosarcoma: a phase II trial from the french sarcoma group (GSF/GETO). *Oncologist*.

[18] Maki R. G., D'Adamo D. R., Keohan M. L. (2009). Phase II study of sorafenib in patients with metastatic or recurrent sarcomas. *Journal of Clinical Oncology*.

[19] Tomita H., Koike Y., Asai M. (2014). Angiosarcoma of the scalp successfully treated with pazopanib. *Journal of the American Academy of Dermatology*.

[20] Miura H., Shirai H. (2015). Low-dose administration of oral pazopanib for the treatment of recurrent angiosarcoma. *Clinical and Experimental Dermatology*.

[21] Kitamura S., Hata H., Imafuku K., Haga N., Homma E., Shimizu H. (2015). Pazopanib can preserve cosmetic quality of life even in end-stage angiosarcoma. *Clinical and Experimental Dermatology*.

[22] van der Graaf W. T. A., Blay J.-Y., Chawla S. P. (2012). Pazopanib for metastatic soft-tissue sarcoma (PALETTE): a randomised, double-blind, placebo-controlled phase 3 trial. *The Lancet*.

[23] Ray-Coquard I. L., Domont J., Tresch-Bruneel E. (2015). Paclitaxel given once per week with or without bevacizumab in patients with advanced angiosarcoma: a randomized phase II trial. *Journal of Clinical Oncology*.

[24] Dickson M. A., D'Adamo D. R., Keohan M. L. (2015). Phase II trial of gemcitabine and docetaxel with bevacizumab in soft tissue sarcoma. *Sarcoma*.

[25] Fraiman G., Ganti A. K., Potti A., Mehdi S. (2003). Angiosarcoma of the small intestine: a possible role for thalidomide?. *Medical Oncology*.

[26] Raina V., Sengar M., Shukla N. K. (2007). Complete response from thalidomide in angiosarcoma after treatment of breast cancer. *Journal of Clinical Oncology*.

[27] Alvarado-Miranda A., Bacon-Fonseca L., Lara-Medina Fernando U., Maldonado-Martínez H., Arce-Salinas C. (2013). Thalidomide combined with neoadjuvant chemotherapy in angiosarcoma of the breast with complete pathologic response: case report and review of literature. *Breast Care*.

[28] Banavali S., Pasquier E., Andre N. (2015). Targeted therapy with propranolol and metronomic chemotherapy combination: sustained complete response of a relapsing metastatic angiosarcoma. *ecancermedicalscience*.

[29] Hsu J.-T., Chen H.-M., Lin C.-Y. (2005). Primary angiosarcoma of the spleen. *Journal of Surgical Oncology*.

[30] Rosner D. (1988). Angiosarcoma of the breast: long-term survival following adjuvant chemotherapy. *Journal of Surgical Oncology*.

[31] Hara T., Tsurumi H., Kasahara S. (2010). Long-term survival of a patient with splenic angiosarcoma after resection, high-dose chemotherapy, and autologous peripheral blood stem cell transplantation. *Internal Medicine*.

